# Differences in proton pumping and Na/H exchange at the leaf cell tonoplast between a halophyte and a glycophyte

**DOI:** 10.1093/aobpla/plu023

**Published:** 2014-05-20

**Authors:** Diana Katschnig, Rinse Jaarsma, Pedro Almeida, Jelte Rozema, Henk Schat

**Affiliations:** 1 Systems Ecology, Department of Ecological Science, Faculty of Earth and Life Sciences, VU University Amsterdam, De Boelelaan 1085, 1081 HV Amsterdam, The Netherlands; 2 Department of Structural Biology, Faculty of Earth and Life Sciences, VU University Amsterdam, De Boelelaan 1085, 1081 HV Amsterdam, The Netherlands; 3 Department of Genetics, Faculty of Earth and Life Sciences, VU University Amsterdam, De Boelelaan 1085, 1081 HV Amsterdam, The Netherlands

**Keywords:** Halophyte, membrane transport, NHX1, *Salicornia*, salt tolerance, vacuole, V-H^+^-ATPase, V-H^+^-PPase.

## Abstract

The tonoplast Na^+^/H^+^ antiporter and tonoplast H^+^ pumps are essential components of salt tolerance in plants. The objective of this study was to investigate the transport activity of the tonoplast Na^+^/H^+^ antiporter and the tonoplast V-H^+^-ATPase and V-H^+^-PPase in a highly tolerant salt-accumulating halophyte, *Salicornia dolichostachya*, and to compare these transport activities with activities in the related glycophyte *Spinacia oleracea*. Vacuolar membrane vesicles were isolated by density gradient centrifugation, and the proton transport and hydrolytic activity of both H^+^ pumps were studied. Furthermore, the Na^+^/H^+^-exchange capacity of the vesicles was investigated by 9-amino-6-chloro-2-methoxyacridine fluorescence. Salt treatment induced V-H^+^-ATPase and V-H^+^-PPase activity in vesicles derived from *S. oleracea*, whereas V-H^+^-ATPase and V-H^+^-PPase activity in *S. dolichostachya* was not affected by salt treatment. Na^+^/H^+^-exchange capacity followed the same pattern, i.e. induced in response to salt treatment (0 and 200 mM NaCl) in *S. oleracea* and not influenced by salt treatment (10 and 200 mM NaCl) in *S. dolichostachya*. Our results suggest that *S. dolichostachya* already generates a high tonoplast H^+^ gradient at low external salinities, which is likely to contribute to the high cellular salt accumulation of this species at low external salinities. At high external salinities, *S. dolichostachya* showed improved growth compared with *S. oleracea*, but V-H^+^-ATPase, V-H^+^-PPase and Na^+^/H^+^-exchange activities were comparable between the species, which might imply that *S. dolichostachya* more efficiently retains Na^+^ in the vacuole.

## Abstract (precis)

The tonoplast Na^+^/H^+^-antiporter and the tonoplast H^+^-pumps are essential components of salt tolerance in plants. We investigated the transport activity of the Na^+^/H^+^-antiporter and the H^+^-pumps in a highly tolerant salt accumulating halophyte, *Salicornia dolichostachya*, and compared them with activities in the related glycophyte *Spinacia oleracea*. Our results suggest that *S. dolichostachya* generates a high tonoplast H^+^-gradient already at low external salinities. At high external salinities, *S. dolichostachya* showed improved growth compared to *S. oleracea,* but H^+^-pump and Na^+^/H^+^-exchange activities were comparable between the species, which might imply that *S. dolichostachya* more efficiently retains Na^+^ in the vacuole.

## Introduction

Salinity negatively affects growth in the vast majority of plant species. These decreases in growth are caused by both the osmotic component of salinity and the direct toxic effects of high levels of Na^+^ inside the plant. Na^+^ can be used as a ‘cheap osmolyte’ in plant adaptation to the low water potential of the saline external environment (Flowers and Colmer 2008). To avoid the toxic effects of salinity, it is crucial for plants to keep cytoplasmic Na^+^ concentrations low. Inside the cytoplasm, Na^+^ negatively interferes with pivotal cellular processes, such as enzyme functioning. The cytoplasmic Na^+^ concentration which is considered toxic is debatable, but likely it is maintained below 200 mM (Flowers and Yeo 1986; Britto and Kronzucker 2010). An important mechanism to keep cytoplasmic Na^+^ concentrations low is compartmentalization of Na^+^ in the vacuole (Carden *et al.* 2003; Kronzucker *et al.* 2006).

Some highly tolerant salt-accumulating halophytes require salt for normal growth and development, and have their growth optimum at external NaCl concentrations between 100 and 300 mM (Flowers and Colmer 2008; Katschnig *et al.* 2013). The fact that high Na^+^ levels, when in the cytoplasm, are detrimental for all plants, including these highly tolerant salt-accumulating halophytes, implies that these halophytes must have evolved enhanced capacities for Na^+^ compartmentalization inside their cells (Flowers and Colmer 2008). It has been estimated that plants maintain their cytoplasmic Na^+^ concentrations below 200 mM (Flowers and Yeo 1986; Britto and Kronzucker 2010), while vacuolar Na^+^ concentrations can be much higher (up to 1200 mM) (Flowers 1985). To maintain osmotic equilibrium within their cells, salt-accumulating halophytes must have evolved, together with an efficient intracellular Na^+^ compartmentalization and retention system, the capacities to synthesize and accumulate compatible solutes in their cytoplasm. Glycophytes, in contrast to salt-accumulating halophytes, show strong growth reductions correlated with increased intracellular Na^+^ concentrations. This implies that intracellular Na^+^ compartmentalization is less successful in glycophytes compared with highly tolerant salt-accumulating halophytes.

The vacuolar Na^+^ compartmentalization capacity may depend on the activity of the Na^+^,K^+^/H^+^ antiporter and/or the steepness of the H^+^ gradient created by one or both of the tonoplast H^+^ pumps. Sequestration of Na^+^ into the vacuole is assumed to be effected by the tonoplast Na^+^,K^+^/H^+^ antiporter (NHX1) (Apse *et al.* 1999; Gaxiola *et al.* 1999), which transports Na^+^ or K^+^, dependent on the prevailing concentration, against the ΔpH into the vacuole (Venema *et al.* 2002). The selectivity of this Na^+^,K^+^/H^+^ antiporter is dependent, besides Na^+^ and K^+^ concentrations, on regulation by the calmodulin-like protein 15, which is in turn dependent on the pH (Yamaguchi *et al.* 2005). The Na^+^,K^+^/H^+^ antiporter uses the energy gradient created by the two tonoplast proton pumps, H^+^-ATPase and H^+^-PPase, to transport Na^+^ into the vacuole. Besides Na^+^ transport into the vacuole, retention of Na^+^ in the vacuole is also likely to be an important mechanism in maintaining low cytoplasmic Na^+^ concentrations (Bonales-Alatorre *et al.* 2013).

The activities of the H^+^ pumps are essential for intracellular Na^+^ sequestration (Flowers and Colmer 2008). However, it is not fully understood if increased sequestration of Na^+^ into the vacuole is achieved by increased activity of V-H^+^-ATPase or V-H^+^-PPase, or both, increased activity of the Na^+^/H^+^ antiporter and/or other mechanisms like, for example, reduced activity of the vacuolar fast- and slow-activating channels (Bonales-Alatorre *et al.* 2013). Increased activity of V-H^+^-ATPase is probably the least likely contributor to increase Na^+^ sequestration into the vacuole (Krebs *et al.* 2010; Shabala 2013). Highly tolerant salt-accumulating halophytes accumulate Na^+^ to very high (1200 mM) intracellular concentrations (Flowers 1985); therefore, they might be useful as model systems to study mechanisms of Na^+^ compartmentalization inside cells. Knowledge about how salt-accumulating halophytes maintain Na^+^ homoeostasis, in comparison with glycophytes, would be useful to increase our current level of understanding of salt tolerance in crop plants.

*Salicornia dolichostachya* is a highly tolerant salt-accumulating halophyte of the Amaranthaceae. It can accumulate up to 400 mM of Na^+^ inside its cells without growth reduction (Katschnig *et al.* 2013). *Salicornia dolichostachya* does not possess any specialized structures for salt storage or removal, such as salt bladders of salt glands. Therefore, it is reasonable to assume that this plant has a high capacity for vacuolar Na^+^ compartmentalization. The family of the Amaranthaceae also contains less salt-tolerant species, such as *Spinacia oleracea.* As the external salt concentration increases, this glycophyte exhibits an increasing accumulation of Na^+^ in its cells, which is accompanied by growth reduction (Robinson *et al.* 1983). Therefore, it can be argued that the capacity to compartmentalize and retain Na^+^ in the vacuole is lower in *S. oleracea* than in *S. dolichostachya*.

Na^+^ transport across the tonoplast membrane can be studied *in vitro* using tonoplast vesicles. This study compared the transport activity of the tonoplast H^+^ pumps: V-H^+^-ATPase and V-H^+^-PPase, and the tonoplast Na^+^/H^+^ antiporter in tonoplast vesicles derived from the highly tolerant salt-accumulating halophyte *S. dolichostachya*, and compared these activities with the transport activities in tonoplast vesicles derived from the related glycophyte *S. oleracea*. Because of the high capacity of *S. dolichostachya* to accumulate Na^+^, we hypothesized that the activity of V-H^+^-PPase or V-H^+^-ATPase, or both, is higher in *S. dolichostachya* than in *S. oleracea*, and this might be accompanied by a higher activity of the Na^+^/H^+^ antiporter.

## Methods

### Plant growth and NaCl treatment

*Salicornia dolichostachya*seeds were collected from a coastal salt marsh at Lutjestrand in The Netherlands. *Spinacia oleracea* seeds were obtained from a commercial supplier (Tuin plus Service, Holland). Seeds of both species were sown on soil (pot soil; Jongkind, Aalsmeer, The Netherlands) and grown for, respectively, 35 days (*S. dolichostachya*) and 16 days (*S. oleracea*). Then seedlings were transferred into individual 1-L polyethylene pots containing a modified half-strength Hoagland solution, composed of (in mM) K^+^, 3; Ca^2+^, 2; Mg^2+^, 0.5; NO_3_^−^, 5; NH_4_^+^, 1.001; HPO_4_^2−^, 1; SO_4_, 0.516; Cl^−^, 0.001; H_2_BO_3_^−^, 0.025; Mn^2+^, 0.002; Zn^2+^, 0.002; Cu^2+^, 0.001; Mo^2+^, 0.001; Fe-Na-EDTA, 0.01, buffered with 2 mM MES, pH 6.0, and in the case of *S. dolichostachya* also 10 mM NaCl. Two weeks after transplanting, NaCl was added in steps of 50 mM day^−1^ to the nutrient solution, and NaCl treatments lasted 8 days after reaching the final salt concentrations. Salt treatments consisted of a control treatment, 0 mM NaCl for *S. oleracea* and 10 mM NaCl for *S. dolichostachya*, and a salt treatment of 200 mM NaCl for both species. *Spinacia oleracea* grows optimally without NaCl in the external medium, while 0 mM NaCl is insufficient to maintain growth in *S. dolichostachya* (Katschnig *et al.* 2013). Therefore, the minimum salt concentration applied to *S. dolichostachya* was 10 mM NaCl. The plants were grown in a randomized block design in a naturally lit greenhouse with additional lamps (photosynthetically active radiation level of the lamps was 250 μmol m^−2^ s^−1^ at plant level, 14/10 h light/dark) in March–May 2013 in Amsterdam, The Netherlands. The average temperature was 20 ± 2/16 ± 2 °C day/night and the relative humidity of the air was 70 ± 10/90 ± 2 % day/night, respectively.

At harvest, six plants per treatment of each species were rinsed with de-mineralized water and carefully blotted dry. These plants were used for biomass, pH and ion content measurements. After rinsing, the plants were separated into shoot and root tissue and weighed, and subsamples of shoot and root tissue were shock frozen in liquid nitrogen and stored at −20 or −80 °C, or oven dried at 70 °C for 72 h. Plant material stored at −20 °C was used to measure pH and oven-dried plant material was used to establish dry mass and ion contents.

For the preparation of tonoplast vesicles, ∼50 g shoot material (several plants combined) per treatment of each species, replicated four times, was excised and immediately homogenized in a blender at 4 °C. The vesicle isolation protocol is described in full in the section Preparation of tonoplast membrane vesicles.

### Tissue Na^+^ and K^+^ concentrations

Oven-dried shoot and root material of *S. oleracea* and *S. dolichostachya* was used to determine Na^+^ and K^+^ contents. The plant material was powdered and heated (∼100 °C) in 5-mL de-mineralized water for 2 h, and thereafter filtered with 4–7-μm cellulose filters (597; Whatman GmbH, Dassel, Germany). Na^+^ and K^+^ contents were determined with a flame atomic spectrophotometer (Perkin-Elmer 1100B; Perkin Elmer Inc., Waltman, MA, USA) and expressed on a cell water basis to obtain the cellular concentrations in mM.

### pH measurements

pH was measured according to the method of Perez-Harguindeguy *et al.* (2013). Frozen (−20 °C) leaf material (0.15 mL) of *S. oleracea* and *S. dolichostachya* was chopped, and shaken in 1.2 mL of demineralized water for 1 h. After shaking, the samples were centrifuged, and the supernatant was used to measure the pH. Measurements were performed with a thin SenTix 41 electrode coupled to an Inolab level 2 pH meter (WTW, Weilheim, Germany).

### Preparation of tonoplast membrane vesicles

Tonoplast membranes were isolated using two-phase partitioning on a sucrose gradient. All steps were carried out at 4 °C and all material was chilled prior to use. Shoot material (∼50 g fresh weight per replicate) of *S. dolichostachya* and *S. oleracea* was homogenized with a blender, immediately after excision, in 100 mL of extraction buffer. There were four independent isolations per treatment per species. The extraction buffer contained 250 mM sucrose, 3 mM MgCl_2_, 100 mM KCl, 2 mM EDTA pH 8, 70 mM TRIS–HCl pH 8, 0.2 % (p/v) polyvinylpyrrolidone (PVPP), 0.1 % (p/v) bovine serum albumin (BSA), 2 mM dithiothreitol (DTT) and cOmplete Protease Inhibitor Tablets (Roche). The homogenate was filtered through four layers of miracloth and centrifuged at 10 000×*g* for 10 min. The pellets were discarded and the supernatant was centrifuged at 100 000×*g* for 60 min. The microsomal pellet was resuspended in resuspension buffer, containing glycerol 15 % (v/v), 1 mM EDTA pH 7.5, 20 mM TRIS–HCl pH 7.5, 1 mM DTT and cOmplete Protease Inhibitor Tablets (Roche). This suspension was layered on top of a discontinuous sucrose gradient consisting of 15 mL of 32 % and 10 mL of 46 % (w/v) sucrose, additionally containing 1 mM EDTA pH 7.5, 20 mM TRIS–HCl pH 7.5 and 1 mM DTT. The sucrose gradient was centrifuged in a swing-out rotor at 80 000×*g* for 3 h. Thereafter, the tonoplast-enriched fraction at the 0/32 % interface was collected, diluted with resuspension buffer and centrifuged at 100 000×*g* for 30 min. The resulting pellet was collected and resuspended in resuspension buffer. Subsequently, 100-µL aliquots were frozen in liquid nitrogen and stored at −80 °C. Protein concentrations were determined by the method of Bradford (1976) using BSA as a standard.

### H^+^-transport assays

The formation and dissipation of a proton gradient across the membranes of vesicles derived from salt-grown (200 mM NaCl) and control-grown (0 mM NaCl in *S. oleracea* and 10 mM NaCl in *S. dolichostachya*) plants was monitored by fluorescence quenching and recovery of 9-amino-6-chloro-2-methoxyacridine (ACMA) upon ATP or pyrophosphate (PP_i_) supply. Reactions were carried out at 22 °C in quartz cuvettes containing 1.5 mL of reaction medium (10 mM MOPS–TRIS pH 7.0, 100 mM KCl, 2 μM ACMA and 2.5 mM MgCl_2_). After addition of the required amount of protein to the reaction medium, cuvettes were placed in the dark chamber of a fluorescence spectrometer (AB2 Luminescence Spectrometer SLM-Aminco, Bowman) and stirred for 5 min preceding the fluorescence readings. Fluorescence was measured at an excitation wavelength of 415 nm and an emission wavelength of 485 nm. When quenching levelled, the formation of the proton gradient by V-H^+^-ATPase or V-H^+^-PPase was initiated by the addition of the required amount of ATP (0.05–1.5 mM) or PP_i_ (0.005–0.25 mM), respectively. Fluorescence quenching was allowed to proceed until a stable value was reached. The initial rate of fluorescence quenching was calculated as a percentage of the rate of fluorescence quenching after dissipation of the ΔpH gradient by 4.5 mM NH_4_Cl at the end of each measurement. We also corrected each measurement for the orientation of the vesicles. Kinetic analysis was performed by fitting a Michaelis–Menten curve through the data points. Curve fitting of the concentration-dependent transport data was performed with non-linear least-squares (nls) model fitting.

### Hydrolytic assays

The rate of ATP hydrolysis of vesicles derived from salt-treated (200 mM NaCl) and control-grown (0 mM NaCl *S. oleracea* and 10 mM NaCl *S. dolichostachya*) plants was determined. To determine the rates of ATP hydrolysis, the release of inorganic phosphate (P_i_) was measured. Sample protein (6 µg) was added to 175 μl of reaction medium. The reaction medium consisted of 50 mM MOPS–TRIS pH 7.0, 250 mM sucrose, 1 mM DTT, 50 mM KCl and 3 mM MgCl_2_. The reaction was started by addition of 75 μL of 3.3 mM ATP (final concentration 1 mM) or 75 μL of 5 mM PP_i_ (final concentration 0.15 mM). After 30-min incubation at 37 °C, reactions were stopped by the addition of 27.5 μL of 30 % (w/w) trichloroacetic acid (TCA) and 30 μL of 10 % sodium dodecyl sulfate (SDS). P_i_ was measured according to the spectrophotometric method of Murphy and Riley (1962). Hydrolytic activity was expressed as nmol P_i_ mg^−1^ protein min^−1^.

### Purity of the tonoplast fraction and ATPase latency tests

The purity of the membrane fractions was estimated by measuring the degree of inhibition of ACMA-fluorescence quenching by 50 mM KNO_3_ and 0.1 mM vanadate (Na_3_VO_4_) to estimate the contributions of vacuolar V-type H^+^-ATPase and plasma membrane P-type H^+^-ATPase, respectively. To assess if the observed fluorescence quenching was due to an H^+^ gradient established over the vesicle membranes and to calculate the initial rate of fluorescence quenching, 4.5 mM NH_4_Cl was added at the end of each measurement.

To establish the sidedness of the vesicles, we measured the release of P_i_ after ATP hydrolysis both with and without 0.06 % (w/v) Triton X-102 in the reaction medium (Meszaros *et al.* 2012). A volume corresponding to 6 µg of protein was added to 175 μL of reaction medium, consisting of 50 mM MOPS–TRIS pH 7.0, 250 mM sucrose, 1 mM DTT, 50 mM KCl and 3 mM MgCl_2_. The reaction was started by addition of 75 μL of 3.3 mM ATP. After 30-min incubation at 37 °C, reactions were stopped by the addition of 27.5 μL of 30 % (w/w) TCA and 30 μL of 10 % SDS. P_i_ was measured according to the spectrophotometric method of Murphy and Riley (1962). The percentage of right-side-out (outside-out) vesicles was calculated as the amount of P_i_ released in the assay without Triton X-102 divided by the amount of P_i_ released in the assay with Triton X-102.

### Na^+^/H^+^-exchange assays

The rate of Na^+^/H^+^ exchange was measured as the dissipation of a pre-established ΔpH gradient. The ΔpH gradient required for identification of Na^+^/H^+^ exchange was generated by V-H^+^-ATPase. The formation of the proton gradient by V-H^+^-ATPase was initiated by the addition of 1 mM ATP. The method and reaction media were the same as described in the section H^+^-transport assays. Fluorescence quenching was allowed to proceed until a stable value was reached, and then different concentrations of NaCl (5–250 mM) were added to the reaction medium. The maximal recovery of fluorescence quenching at 180 s after NaCl addition was recorded. The rate of Na^+^/H^+^ exchange was expressed as a percentage of the initial quenching.

### Statistical analysis

Normality and homogeneity of the data were checked. Student's *t*-test was used to assess the effect of salt treatment on ash-free dry mass and shoot Na^+^ and shoot K^+^ accumulation. One-way ANOVAs with Tukey's *post hoc* test were used to assess the effect of salt treatment and species on shoot pH, hydrolytic activity of V-H^+^-ATPase and tonoplast Na^+^/H^+^-exchange capacity, and two-way ANOVAs were used to examine the species × salt treatment interaction effect on these parameters. Kinetic parameters with standard errors of the ATP-dependent and PP_i_-dependent H^+^-translocation activity were calculated with nls model fitting in R (R Core Team 2012).

## Results

### Characterization of the plant material

#### Plant ash-free dry mass

**Figure 1.**
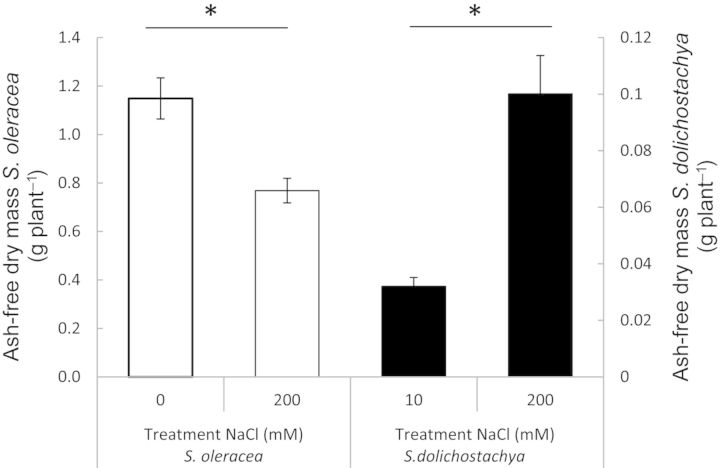
Ash-free dry mass of *Spinacia oleracea* and *Salicornia dolichostachya* after 8 days of growth. Plants were exposed for 8 days to 0 and 200 mM NaCl for *S. oleracea*, and 10 and 200 mM NaCl for *S. dolichostachya*. Values shown are means ± SEM with six replicate plants per treatment. Note that the ordinates of the two species have different scales. The treatment effect was statistically significant for both species (Student's *t*-test, *P < 0.05).

Salt treatment had a negative effect on growth of *S. oleracea* and a positive effect on growth of *S. dolichostachya* (Fig. 1). Ash-free dry mass was, as a result of the salt treatment, reduced by ∼30 % in *S. oleracea* and approximately tripled in *S. dolichostachya* compared with the control treatment.

#### Shoot Na^+^ and K^+^ concentrations

**Figure 2.**
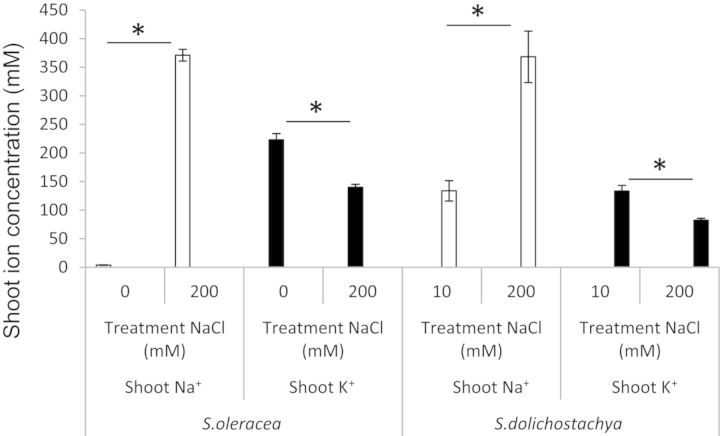
Na^+^ and K^+^ concentrations in the shoot of *Spinacia oleracea* and *Salicornia dolichostachya* after 8 days of growth. Plants were exposed for 8 days to 0 and 200 mM NaCl for *S. oleracea*, and 10 and 200 mM NaCl for *S. dolichostachya*. Values shown are means ± SEM with six replicate plants per treatment. The treatment effect was statistically significant for shoot Na^+^ and shoot K^+^ concentrations for both species (Student's *t*-test, *P < 0.05).

Na^+^ concentrations were comparable in *S. oleracea* and *S. dolichostachya* after 8 days of growth in 200 mM NaCl; however, at low external salinity Na^+^ concentrations were much higher in *S. dolichostachya* than in *S. oleracea* (Fig. 2)*.* K^+^ concentrations decreased with salt treatment in both *S. oleracea* and *S. dolichostachya*.

#### Shoot pH

**Figure 3.**
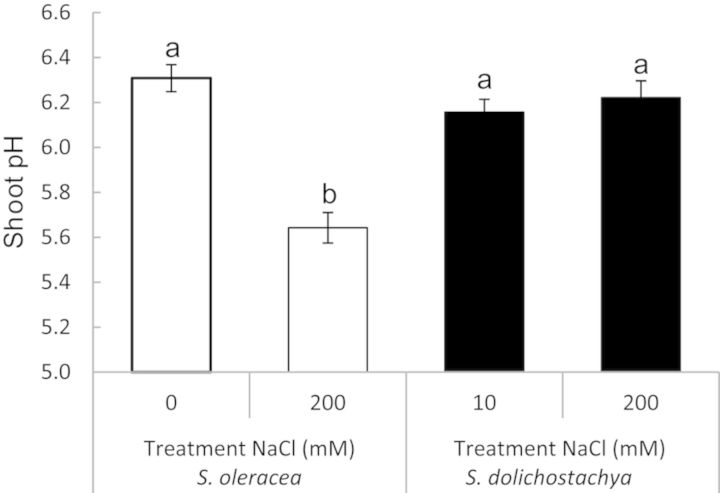
Shoot pH of *Spinacia oleracea* and *Salicornia dolichostachya* after 8 days of growth. Plants were exposed for 8 days to 0 and 200 mM NaCl for *S. oleracea*, and 10 and 200 mM NaCl for *S. dolichostachya*. Values shown are means ± SEM with six replicate plants per treatment. Different letters indicate a statistically significant treatment effect on leaf pH (one-way ANOVA, *P* < 0.05). Interaction effect: two-way ANOVA, species×treatment *P* < 0.05.

Salinity decreased shoot pH by ∼0.6 units in *S. oleracea*, but had no effect on shoot pH in *S. dolichostachya* (Fig. 3).

### Characterization of the membrane fractions

**Figure 4.**
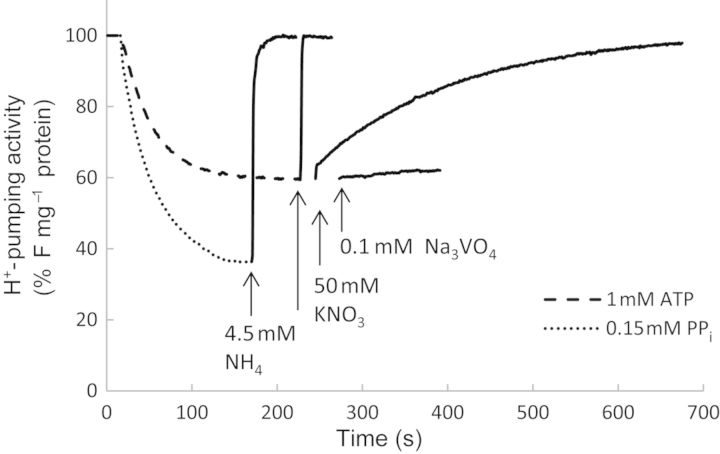
Characteristics of the H^+^-ATPase- and H^+^-PPase-dependent ΔpH gradient. H^+^ translocation was determined by measuring the level of fluorescence quenching of ACMA. Formation of the ΔpH gradient was initiated by adding 1 mM ATP or 0.15 mM PP_i_. When ATP-dependent fluorescence quenching reached a stable value, 50 mM KNO_3_ or 0.1 mM Na_3_VO_4_ was added. Five micrograms of protein was used per assay. Recordings of vesicles derived from shoots of 200 mM grown *Spinacia oleracea* are shown.

H^+^-translocating activity and hydrolytic activity of V-H^+^-ATPase and V-H^+^-PPase were determined in vesicles derived from shoots of salt-treated (200 mM NaCl) and control-grown *S. dolichostachya* (10 mM NaCl) and *S. oleracea* (0 mM NaCl). To characterize the V-H^+^-ATPase and V-H^+^-PPase activities in *S. dolichostachya* and *S. oleracea*, the hydrolysis of ATP and the magnitude of ATP- and PP_i_-dependent ΔpH formation were determined. The formation of a transmembrane H^+^ gradient was measured by fluorescence quenching of the surface charge density probe ACMA. After addition of ATP or PP_i_, fluorescence quenching was recorded and allowed to proceed until it reached a stable value. Addition of NH_4_Cl resulted in an instantaneous collapse of the ΔpH gradient and full recovery of the fluorescence signal (Fig. 4). Recovery of the fluorescence signal by the uncoupler NH_4_Cl demonstrated that fluorescence quenching was induced by an established ΔpH gradient generated by H^+^ translocation of H^+^-ATPase and H^+^-PPase.

The purity of the tonoplast fractions was estimated by measuring the recovery of a pre-established ΔpH gradient in the presence of inhibitors that differentiate specific types of H^+^-ATPases. V-H^+^-ATPase is sensitive to nitrate and insensitive to vanadate (Na_3_VO_4_). Vanadate is an inhibitor of the P-type H^+^-ATPase. V-H^+^-ATPase activity, measured as nitrate-sensitive and vanadate-insensitive H^+^-translocation activity, was observed in membrane fractions collected at the 0/32 % interface of the continuous sucrose gradient. In these fractions, the recovery of the pre-established ΔpH gradient generated by V-H^+^-ATPase after addition of vanadate was small, and addition of nitrate led to ∼95 % recovery of the fluorescence signal (Fig. 4). Membrane fractions isolated from *S. dolichostachya* and *S. oleracea* showed similar fluorescence-recovery responses in the presence of the inhibitors. Moreover, the degrees of inhibition were similar in salt-treated and control plants, indicating that the salt treatment did not affect the composition of the isolated membrane fractions.

Only tonoplast vesicles with an outside-out orientation (cytoplasmic-side out) participate in H^+^-ATPase- and H^+^-PPase-dependent transport. Therefore, we determined the percentage of outside-out-orientated vesicles by measuring the hydrolytic activity of V-H^+^-ATPase in medium with and without the detergent Triton X-102. The percentage of outside-out vesicles was 57 % ± 3.2 and 54.3 % ± 1.0 in *S. oleracea*-derived vesicles of 0 and 200 mM NaCl-treated plants, respectively, and 33.3 % ± 3.6 and 31.4 % ± 2.2 in *S. dolichostachya*-derived vesicles of 10 and 200 mM NaCl-treated plants, respectively (Table 1). Because of the differences in orientation of the isolated membrane fractions in *S. dolichostachya* and *S. oleracea*, we corrected all the H^+^-translocation activity and hydrolytic activity values of V-H^+^-ATPase and V-H^+^-PPase for the percentage of outside-out-orientated vesicles.

**Table 1.** H^+^-ATPase activity, measured as release of P_i_, of tonoplast vesicles derived from shoots of *Spinacia oleracea* or *Salicornia dolichostachya* and grown with 200 mM NaCl or control salinity (0 mM NaCl for *S. oleracea* and 10 mM NaCl for *S. dolichostachya*) in the external medium. The vesicles were intact (−Triton X-102) or collapsed by 0.06 % (w/v) Triton X-102 (+Triton X-102). Values shown are means ± SEM with four replicates per treatment. Different letters indicate a statistically significant effect on orientation of the vesicles between the four different treatment–species combinations (one-way ANOVA, *P*< 0.05).

Species	Treatment NaCl (mM)	ATPase activity (nmol P_i_ mg protein^−1^ m^−1^)		Outside-out (%)
		−Triton X-102 ± SEM	+Triton X-102 ± SEM	
*S. oleracea*	0	1129.9 ± 109.0	2012.9 ± 196.2	57.0 ± 3.2a
	200	1676.1 ± 92.3	3089.2 ± 151.4	54.3 ± 1.0a
*S. dolichostachya*	10	1016.5 ± 142.4	3009.8 ± 194.3	33.3 ± 3.6b
	200	842.4 ± 54.5	2726.3 ± 184.3	31.4 ± 2.2b

### Na^+^/H^+^ exchange

**Figure 5.**
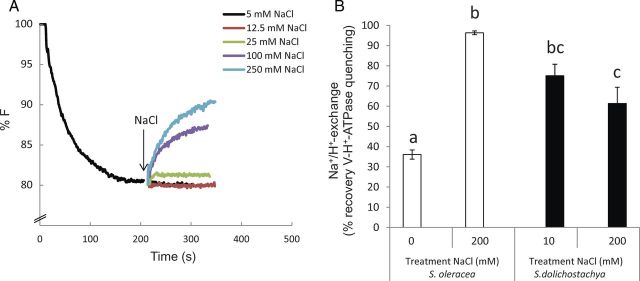
(A) Na^+^/H^+^-exchange capacity of membrane vesicles derived from shoots of the halophyte *Salicornia dolichostachya* grown at 200 mM NaCl. The Na^+^/H^+^-exchange capacity of the vesicles was measured as the level of recovery of ACMA-fluorescence quenching of a pre-established ΔpH gradient created by V-H^+^-ATPase after 1 mM ATP addition. In total, 2.5 μg of protein was used per assay. (B) Na^+^/H^+^-exchange capacity of membrane vesicles derived from shoots of the halophyte *Salicornia dolichostachya* and the glycophyte *Spinacia oleracea*. The Na^+^/H^+^-exchange capacity of the vesicles was measured as the level of recovery of ACMA-fluorescence quenching by addition of 250 mM NaCl of a pre-established ΔpH gradient created by V-H^+^-ATPase. Plants were grown under control conditions (10 mM NaCl for *S. dolichostachya* and 0 mM NaCl for *S. oleracea*) or saline conditions (200 mM NaCl for both species) for 8 days. Data points are means ± SEM of three independent membrane isolations per treatment. Different letters indicate a statistically significant treatment effect on Na^+^/H^+^-exchange capacity (one-way ANOVA, *P* < 0.05). Interaction effect: two-way ANOVA, species×treatment *P* < 0.05.

The effect of Na^+^ (range 0–250 mM) on the dissipation of a pre-established ΔpH gradient created by V-H^+^-ATPase was tested in tonoplast membrane vesicles derived from shoots of salt-grown (200 mM NaCl) and control-grown *S. dolichostachya* (10 mM NaCl) and *S. oleracea* (0 mM NaCl). The dissipation of the pre-established ΔpH gradient was measured as recovery of the ACMA-fluorescence signal. Addition of NaCl resulted in dissipation of the pre-established H^+^ gradient. Additions of higher concentrations of Na^+^ resulted in a stronger recovery of the fluorescence signal (Fig. 5A). When ATP was omitted from the reaction medium, Na^+^ uptake was very low and independent of vesicle origin (data not shown).

Addition of 250 mM NaCl in vesicles derived from 200 mM NaCl-grown *S. oleracea* resulted in recovery of the fluorescence signal to approximately the level observed before ATP addition (Fig. 5B). The level of recovery of the fluorescence signal was significantly lower in 0 mM NaCl-grown *S. oleracea* than in 200 mM NaCl-grown *S. oleracea.* Na^+^/H^+^ exchange in *S. dolichostachya* did not differ between vesicles derived from salt-treated (200 mM NaCl) and non-salt-treated (10 mM NaCl) plants.

### Activity of the V-H^+^-ATPase and V-H^+^-PPase of *S. dolichostachya* and *S. oleracea* in response to salt treatment

**Figure 6.**
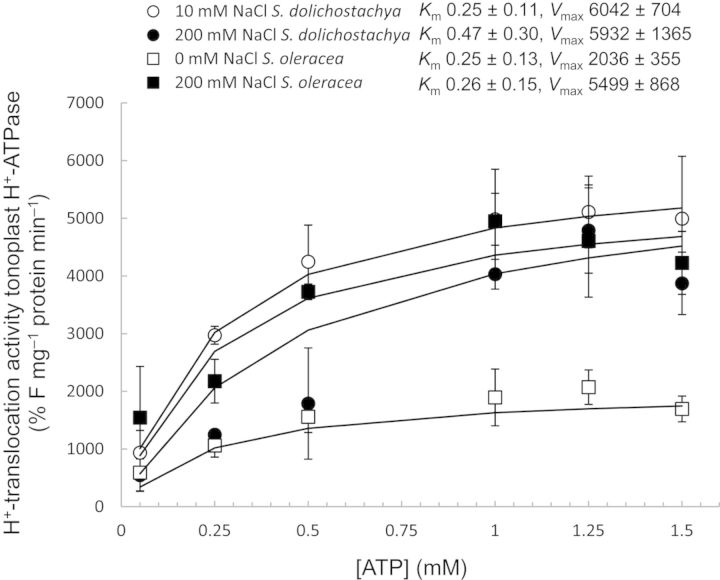
H^+^-translocation activity of the tonoplast V-H^+^-ATPase of vesicles derived from shoots of the halophyte *Salicornia dolichostachya* (circles) and the glycophyte *Spinacia oleracea* (squares). The H^+^-translocation activity of V-H^+^-ATPase was determined by measuring the level of fluorescence quenching of ACMA. Plants were grown under control conditions (10 mM NaCl for *S. dolichostachya* and 0 mM NaCl for *S. oleracea*, open symbols) or saline conditions (200 mM NaCl for both species, closed symbols) for 8 days. Data points are means ± SEM of three independent membrane isolations per treatment. Kinetic analysis was performed by fitting a Michaelis–Menten curve through the data points. Kinetic parameters with standard errors were calculated with nls model fitting in R.

**Figure 7.**
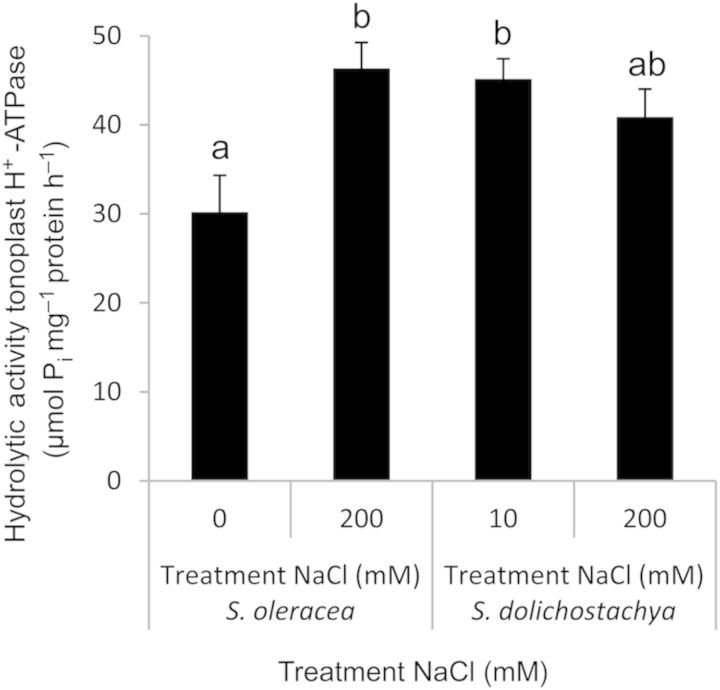
Hydrolytic activity of the tonoplast V-H^+^-ATPase of vesicles derived from shoots of the halophyte *Salicornia dolichostachya* and the glycophyte *Spinacia oleracea*. The hydrolytic activity of V-H^+^-ATPase was determined by measuring the amount of inorganic phosphate (P_i_) released. Plants were grown under control conditions (10 mM NaCl for *S. dolichostachya* and 0 mM NaCl for *S. oleracea*) or saline conditions (200 mM NaCl for both species) for 8 days. Data points are means ± SEM of four independent membrane isolations per treatment. Different letters indicate a statistically significant treatment effect on hydrolytic activity (one-way ANOVA, *P* < 0.05). Interaction effect: two-way ANOVA, species×treatment *P* < 0.05.

The H^+^-translocating activity of V-H^+^-ATPase in vesicles derived from *S. oleracea*, measured as the rate of fluorescence quenching in the first 120 s following the addition of ATP, was ∼2-fold higher in salt-grown (200 mM NaCl) than in control-grown (0 mM NaCl) plants (Fig. 6). The hydrolytic activity of V-H^+^-ATPase, measured as the release of P_i_, showed the same pattern as the H^+^-translocating activity; it was higher in salt-grown *S. oleracea* (200 mM NaCl) than in control-grown *S. oleracea* (0 mM NaCl) (Fig. 7). In contrast to *S. oleracea*, the H^+^-translocating activity of V-H^+^-ATPase in vesicles derived from *S. dolichostachya* did not differ between salt-grown (200 mM NaCl) and control-grown (10 mM NaCl) plants (Fig. 6). Accordingly, the hydrolytic activity of V-H^+^-ATPase showed the same pattern as the H^+^-translocating activity in that it did not differ between vesicles derived from salt-grown (200 mM NaCl) and control-grown (10 mM NaCl) *S. dolichostachya* (Fig. 7).

**Figure 8.**
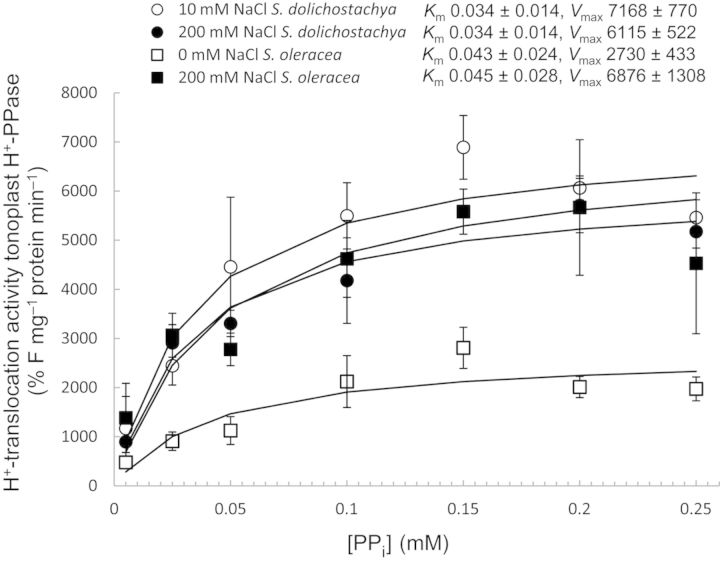
H^+^-translocation activity of the tonoplast V-H^+^-PPase of vesicles derived from shoots of the halophyte *Salicornia dolichostachya* (circles) and the glycophyte *Spinacia oleracea* (squares). The H^+^-translocation activity of V-H^+^-PPase was determined by measuring the level of fluorescence quenching of ACMA. Plants were grown under control conditions (10 mM NaCl for *S. dolichostachya* and 0 mM NaCl for *S. oleracea*, open symbols) or saline conditions (200 mM NaCl for both species, closed symbols) for 8 days. Data points are means ± SEM of three independent membrane isolations per treatment. Kinetic analysis was performed by fitting a Michaelis–Menten curve through the data points. Kinetic parameters with standard errors were calculated with nls model fitting in R.

The H^+^-translocating activity of V-H^+^-PPase in vesicles derived from both *S. oleracea* and *S. dolichostachya* followed the same pattern as the H^+^-translocating activity of V-H^+^-ATPase. Vesicles derived from salt-grown (200 mM NaCl) *S. oleracea* had a greater H^+^-translocating activity than vesicles derived from control-grown (0 mM NaCl) plants, and, in contrast to *S. oleracea*, the H^+^-translocating activity of V-H^+^-PPase in vesicles derived from *S. dolichostachya* did not differ between salt-grown (200 mM NaCl) and control-grown (10 mM NaCl) plants (Fig. 8).

## Discussion

Since high concentrations of Na^+^ (>200 mM) in the cytoplasm are assumed to be toxic, the storage of Na^+^ is assumed to be restricted to the vacuole (Flowers and Yeo 1986). Vacuolar H^+^ pumps and the vacuolar Na^+^,K^+^/H^+^ antiporter are essential for Na^+^ sequestration into the vacuole. Over-expression of the vacuolar Na^+^,K^+^/H^+^ antiporter NHX1 has been argued to increase the salt tolerance of transgenic plants (Apse *et al.* 1999; Zhang and Blumwald 2001), which propelled NHX1 as a promising target to improve salt tolerance in plants. However, the reported effects of over-expression of NHX1 on salt tolerance in transgenic plants are far from impressive thus far (Rozema *et al.* 2013). Therefore, to what extent the tonoplast Na^+^,K^+^/H^+^-exchange capacity differs between a highly salt-tolerant salt-accumulating halophyte such as *S. dolichostachya* and a related glycophyte is interesting.

We investigated Na^+^/H^+^-exchange activity in tonoplast membrane vesicles derived from shoots of *S. dolichostachya*, and compared these activities to activities in tonoplast membrane vesicles derived from shoots of the related glycophyte *S. oleracea.* Dissipation of the ΔpH gradient after addition of 200 mM NaCl (Fig. 5B) was the same in vesicles derived from both control-grown (10 mM NaCl) and salt-treated (200 mM NaCl) *S. dolichostachya*. In contrast, dissipation of the ΔpH gradient in *S. oleracea* was influenced by the salt treatment: the dissipation was higher in vesicles derived from salt-treated (200 mM NaCl) *S. oleracea* than in control-grown *S. oleracea* (0 mM NaCl). This salt-induced stimulation of the Na^+^–H^+^ exchange, as observed in *S. oleracea*, has often been reported for glycophytic species (Vera-Estrella *et al.* 2005; Queiros *et al.* 2009). In some species, no activity of the tonoplast Na^+^/H^+^ exchanger could be detected at all (*Medicago media*; Staal *et al.* 1991), or only after exposure to salinity (Garbarino and Dupont 1988). The inducibility of the tonoplast Na^+^/H^+^ exchanger might be related to stress perception. *Salicornia oleracea* does experience stress at the salt concentration used for salt treatment in this experiment (200 mM NaCl), as can be seen by the reduction in biomass in this species (Fig. 1). A growth reduction in *S. oleracea* in response to salt treatment is already apparent at low (10 mM NaCl) external salinity (D. Katschnig unpubl. res.). Low external Ca^2+^ concentrations can cause a decline in cytoplasmic K^+^ concentrations (Shabala *et al.* 2006), and since we did not keep the Na^+^/Ca^2+^ ratio constant while applying salt treatment, the effect of NaCl in our experiments might have been more severe than if a constant Na^+^/Ca^2+^ ratio had been used. In contrast to *S. oleracea*, *S. dolichostachya* does not experience salt stress at external salinities lower and equal to its growth optimum, but is stimulated in its growth by Na^+^. The high activity of the tonoplast Na^+^/H^+^ exchanger in *S. dolichostachya* that we observed in this study is consistent with the observation that rates of Na^+^ accumulation in halophytic species can be extremely high, even under low levels of salt exposure (Flowers and Yeo 1986; Cheeseman 1988). Therefore, it is likely that the high activity of the tonoplast Na^+^/H^+^ exchanger in *S. dolichostachya* in response to 10 mM NaCl treatment reflects the constitutive Na^+^ accumulation in this species. Our results are in line with the conclusion of Shabala and Mackay (2011) that tonoplast Na^+^/H^+^ exchangers are often constitutive in halophytes and inducible by salt treatment in salt-tolerant glycophytes (Shabala and Mackay 2011). However, the Na^+^/H^+^ exchanger has also been reported to be induced by 200 mM NaCl (control treatment 5 mM NaCl) in a species with salt tolerance and salt accumulation strategies comparable to *S. dolichostachya* (*Salicornia bigelovii*; Parks *et al.* 2002). It is possible that the difference in inducibility of the Na^+^/H^+^ exchanger observed between the study of Parks *et al.* (2002) and our study is caused by the differences in control salinity. It might be that the 10 mM NaCl treatment was enough to cause an induction of the activity of the tonoplast Na^+^/H^+^ exchanger in *S. dolichostachya*, and that 10 mM NaCl treatment would cause an induction of the Na^+^/H^+^ exchanger in *S. oleracea.* However, because *S. oleracea*, in contrast to *S. dolichostachya*, accumulates very low concentrations (<10-fold) of Na^+^ in its leaves in response to 10 mM NaCl treatment (D. Katschnig unpubl. res.), a comparable induction of the Na^+^/H^+^ exchanger between the two species is not likely.

The ΔpH needed for Na^+^ transport over the tonoplast membrane is generated by V-H^+^-ATPase and V-H^+^-PPase (Apse *et al.* 1999; Gaxiola *et al.* 2007). In our study, both the H^+^-translocating activity and the hydrolytic activity of V-H^+^-ATPase (Figs 6 and 7) followed the same pattern in response to salinity as the Na^+^/H^+^-exchange activities (Fig. 5B), both were upregulated in *S. oleracea*, whereas they were high and not influenced by salt treatment in *S. dolichostachya*. The inducibility of the H^+^ pumps in *S. oleracea* and the constitutive activity of the H^+^ pumps in *S. dolichostachya* in response to salt treatment were reflected in the total shoot pH of the two species. Salt treatment decreased the total shoot pH in *S. oleracea*, whereas it did not affect the total shoot pH in *S. dolichostachya* (Fig. 3). Therefore, the total shoot pH seems to reflect changes in the vacuolar pH, such as previously demonstrated for the total petal pH in *Petunia hybrida* (Quattrocchio *et al.* 2006). Salt-induced H^+^-translocating activity or hydrolytic activity of the V-H^+^-ATPase has been reported for several glycophytic and halophytic species (Ayala *et al.* 1996; Barkla *et al.* 2002; Vera-Estrella *et al.* 2005). Also protein levels and transcript levels of subunits of the gene coding for V-H^+^-ATPase have been reported to increase in response to NaCl treatment (Golldack and Dietz 2001; Ratajczak *et al.* 1994). However, such effects were not found in other studies (cf. Binzel and Ratajczak 2002). In studies of mutants, Krebs *et al.* (2010) found that V-H^+^-ATPase is not involved in vacuolar Na^+^ sequestration in gamete and embryo development in *Arabidopsis thaliana* (Krebs *et al.* 2010). In addition, Bose *et al.* (2014) argued that the ATP pool is greatly reduced under saline conditions due to the restoration of an otherwise depolarized plasma membrane potential and because of the synthesis of organic osmolytes (Bose *et al.* 2014), which implies that V-H^+^-PPase will be the main generator of the H^+^ gradient needed for proper functioning of the tonoplast Na^+^–H^+^ exchanger under saline conditions (Shabala 2013; Bose *et al.* 2014). In our experiment, the H^+^-translocating activity of V-H^+^-PPase also followed, just like that of V-H^+^-ATPase, the same pattern as we observed for the tonoplast Na^+^/H^+^ exchanger (Fig. 8). Literature reports on the inducibility of the tonoplast V-H^+^-PPase are highly inconsistent (cf. Silva and Geros 2009). V-H^+^-PPase activity is sometimes reported to increase (Wang *et al.* 2001; Queiros *et al.* 2009), but as often reported to decrease (Wang *et al.* 2000; Otoch *et al.* 2001), in response to salt treatment. V-H^+^-PPase is dependent on K^+^ for its activation (Rea and Poole 1993). Under saline conditions, K^+^ concentrations in the cytoplasm can become low (Cuin *et al.* 2003; Shabala and Cuin 2007), which would have a negative effect on the functioning of V-H^+^-PPase. Thus efficient K^+^ retention inside the cytoplasm is needed to enable the functioning of H^+^-PPase, which makes efficient K^+^ retention a necessity for transgenic plants over-expressing the Na^+^/H^+^ exchanger (Shabala 2013). This might also explain the low success of increasing salt tolerance in transgenic plants over-expressing the vacuolar Na^+^,K^+^/H^+^ antiporter NHX1 (Shabala 2013).

Vesicles derived from both *S. dolichostachya* and *S. oleracea* had a similar activity of V-H^+^-ATPase, V-H^+^-PPase and the Na^+^/H^+^ exchanger when treated with 200 mM NaCl (Figs 5–8). Based on these results, it seems unlikely for *S. oleracea* to be limited in its Na^+^/H^+^-transport capacity over the tonoplast. However, *S. dolichostachya* had better growth compared with *S. oleracea* when treated with 200 mM NaCl, which could imply that Na^+^ compartmentalization is more efficient in *S. dolichostachya* than in *S. oleracea*. Efficient Na^+^ compartmentalization is not only dependent on Na^+^ transport into the vacuole, but also on the rate of Na^+^ leakage from the vacuole into the cytoplasm. Since salt treatment had comparable effects on Na^+^–H^+^-transport activities in *S. dolichostachya* as in *S. oleracea*, it is possible that *S. dolichostachya* more efficiently retains Na^+^ within the vacuole. Retention of Na^+^ within the vacuole has been correlated with structural membrane traits such as lipid composition (Leach *et al.* 1990), but, more likely, also the tonoplast Na^+^-permeable channels could play a major part in Na^+^ retention inside the vacuole. This is illustrated by the finding that regulation of the slow- and fast-activating vacuolar channels is fundamental for salinity tolerance in *Chenopodium quinoa* (Bonales-Alatorre *et al.* 2013). Next to less efficient Na^+^ retention inside the vacuole, it is also possible that *S. oleracea*, compared with *S. dolichostachya*, has a lower ability of synthesizing and accumulating compatible solutes in its cytoplasm. To maintain water potential equilibrium within the cell, compatible solutes are assumed to accumulate in the cytoplasm when Na^+^ is stored in the vacuole (Storey and Wynjones 1979). Thus, the differences in growth between *S. oleracea* and *S. dolichostachya* at 200 mM NaCl cannot be explained by differences in Na^+^/H^+^-exchange activity or H^+^-pumping activity, which suggests that Na^+^ retention inside the vacuole is stronger in *S. dolichostachya* compared with *S. oleracea* or that its capacity to maintain water potential equilibrium within the cell is higher.

## Conclusions

Our results showed that in response to salt treatment, the activity of V-H^+^-ATPase, V-H^+^-PPase and also the Na^+^/H^+^ exchanger was induced in *S. oleracea* (0 and 200 mM NaCl treatment), whereas in *S. dolichostachya* these activities were high and not induced by salt treatment (10 and 200 mM NaCl treatment). This might reflect the fact that *S. dolichostachya* is an obligate halophyte with a constitutive salt requirement. Furthermore, *S. dolichostachya* grew better compared with *S. oleracea* when treated with 200 mM NaCl, but both species had a similar capacity for Na^+^ influx into the vacuole. This might be taken as an indication that *S. dolichostachya* had a more efficient system of Na^+^ retention inside the vacuole than *S. oleracea*. It could also suggest that the water potential equilibrium within the cell was better regulated in *S. dolichostachya* than in *S. oleracea*.

## Sources of Funding

This work was carried out within the PhD project of D.K., and was supported by Project 2.3.2: ‘Adaptation to dry and saline conditions by crop cultivation exploiting brackish water and saving fresh water’ of the Dutch National Research Program: Knowledge for Climate and with co-funding of Project ZKK-1 of the Zilte Kennis Kring.

## Contributions by the Authors

D.K. designed the experiment. D.K., R.J. and P.A. carried out the experiment. D.K. analysed the results. D.K. and H.S. wrote the manuscript. All authors read and approved the final manuscript.

## Conflict of Interest Statement

None declared.
